# Demonstration of a chip-based optical isolator with parametric amplification

**DOI:** 10.1038/ncomms13657

**Published:** 2016-11-25

**Authors:** Shiyue Hua, Jianming Wen, Xiaoshun Jiang, Qian Hua, Liang Jiang, Min Xiao

**Affiliations:** 1 National Laboratory of Solid State Microstructures, College of Engineering and Applied Sciences, and School of Physics, Nanjing University, Nanjing 210093, China; 2 Department of Applied Physics, Yale University, New Haven, Connecticut 06511, USA; 3 Synergetic Innovation Center in Quantum Information and Quantum Physics, University of Science and Technology of China, Hefei, Anhui 230026, China; 4 Department of Physics, University of Arkansas, Fayetteville, Arkansas 72701, USA

## Abstract

Despite being fundamentally challenging in integrated (nano)photonics, achieving chip-based light non-reciprocity becomes increasingly urgent in signal processing and optical communications. Because of material incompatibilities in conventional approaches based on the Faraday effect, alternative solutions have resorted to nonlinear processes to obtain one-way transmission. However, dynamic reciprocity in a recent theoretical analysis has pinned down the functionalities of these nonlinear isolators. To bypass such dynamic reciprocity, we here demonstrate an optical isolator on a silicon chip enforced by phase-matched parametric amplification in four-wave mixing. Using a high-*Q* microtoroid resonator, we realize highly non-reciprocal transport at the 1,550 nm wavelength when waves are injected from both directions in two different operating configurations. Our design, compatible with current complementary metal-oxide-semiconductor (CMOS) techniques, yields convincing isolation performance with sufficiently low insertion loss for a wide range of input power levels. Moreover, our work demonstrates the possibility of designing chip-based magnetic-free optical isolators for information processing and laser protection.

## Abstract (web summary)

Non-reciprocal optical elements usually require the presence of magnetic fields, which makes chip integration difficult. Here, Hua *et al*. demonstrate a non-magnetic optical isolator with bidirectional injection on a silicon platform utilizing parametric amplification in four-wave mixing.

## Graphical abstract

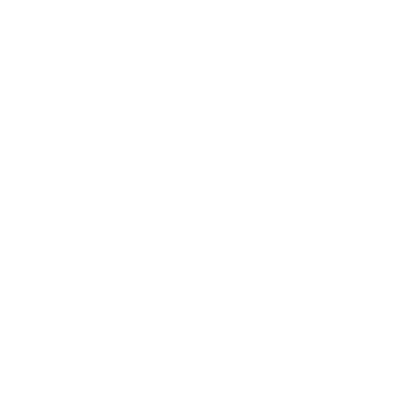

Non-reciprocal photonic devices^1^ that break time-reversal symmetry provide crucial functionalities such as isolation and circulation in laser protection, optical signal processing and instrumentation applications. Yet, reciprocity, as constrained by the Lorentz theorem^2^, is fundamental to light transport in linear, time-invariant optical systems and holds even in rather complex ones. Although widely used in optical communications and sensing, non-reciprocal devices are still challenging in silicon integrated photonics owing to limitations in material integration and device design. To break the reciprocity, a traditional approach is to guide light through materials with a strong magneto-optical Faraday effect^3^,^4^. Regardless of its versatility, this approach usually encounters severe obstruction from the miniaturization of bulky volumes and material compatibility with mature integrated silicon photonic platforms. Although small footprint^5^,^6^ could be obtained with advanced bonding and deposition technologies, the application of an external magnetic field may deleteriously interfere with nearby optics and influence their functionalities.

The quest for alternative and more compact isolation schemes has recently garnered an immense impetus and spawned a variety of methods by adopting different physical principles to avoid the need for the integration of magneto-optical elements. In one alternative direction, a notable effort has been made upon reproducing the effect of magneto optics using non-magnetic structures undergoing spatiotemporal modulations^7^,^8^,^9^,^10^,^11^ (an idea akin to the one used a while ago for non-reciprocal mode conversion in optical fibres^12^). In spite of the conceptual elegance, unfortunately, most of the reported systems^8^,^9^,^10^,^11^ to date have to rely on complex structures, which demand operating thresholds often with fairly low non-reciprocal outputs. In contrast to linear isolators, considerable enthusiasm has been devoted to breaking Lorentz reciprocity with use of various nonlinearities. Among these, to name a few, non-reciprocal light transmission has recently been demonstrated with second-order nonlinearity^13^,^14^, Kerr or Kerr-type nonlinearities^15^,^16^, gain/absorption saturation^17^,^18^,^19^, Raman amplification^20^, stimulated Brillouin scattering^21^, Bragg scattering^22^, thermo-optic effects^23^ and with opto-acoustic effects^24^.

Of these nonlinear means^13^,^14^,^15^,^16^,^17^,^18^,^19^,^20^,^21^,^22^,^23^,^24^, asymmetric transmission contrast is typically demonstrated when a wave is injected in either forward or backward direction but never both. The lack of an experiment on the simultaneous presence of waves from both directions causes people to question whether these nonlinear isolators could be actually capable of providing complete isolation under practical operating conditions. In a very recent theoretical work, fortunately, this hypothesis has been partially disproved by Shi and his co-workers^25^. Specifically, they found that for Kerr or Kerr-like nonlinearities, due to the existence of a dynamic reciprocity, nonlinear isolators of this type fail to show any isolation for arbitrary backward-propagating noise coexisting with a forward signal. Moreover, their results point out an important limitation on the use of nonlinear optical isolators for signal processing and laser protection. The discovery on dynamic reciprocity further prompts these authors to query whether such a property is generally accompanied by a nonlinear optical isolator. It is therefore fundamentally intriguing to know whether nonlinear means could be chosen to construct a real chip-based optical isolator for practical applications, especially for laser protection.

To give an affirmative answer, here we propose and demonstrate the realization of a chip-based, non-magnetic optical isolator in a high-*Q* silica microtoroid resonator^26^ by exploiting phase-matched parametric amplification in four-wave mixing. In comparison with previous works^13^,^14^,^15^,^16^,^17^,^18^,^19^,^20^,^21^,^22^,^23^,^24^ utilizing nonlinearities, our framework shows explicitly remarkable isolating performance in suppressing the transmission of backward-propagating noise with signal fields launched from both directions simultaneously. Unlike prior work^17^,^21^,^23^, our scheme achieves appreciable isolation functionality with only one coupling fibre, a significant non-trivial step forward. Because our device is compatible with mature complementary metal-oxide-semiconductor (CMOS) techniques, it paves the way for realizing practical on-chip optical isolators through nonlinear processes beyond the constraint posed by dynamic reciprocity.

## Results

### Basic principles

To bypass the dynamic reciprocity, our work explores phase-matched parametric amplification in four-wave mixing (Supplementary Note 1). Imposed by the phase matching within the microcavity, the forward input signal experiences parametric amplification, while the backward input remains almost intact owing to the lack of suitable phase matching. Because of the inversion symmetry of silica, the dominant parametric process is four-wave mixing through third-order nonlinearity. In the process, two pump photons (with angular frequency *ω*_p_ and wave vector **k**_**p**_) are converted to one signal photon (*ω*_s_, **k**_**s**_) plus one idler (*ω*_i_, **k**_**i**_) by satisfying the phase-matching condition: 2*ω*_p_=*ω*_s_+*ω*_i_ (energy conservation) and 2**k**_**p**_=**k**_**s**_+**k**_**i**_ (momentum conservation). It is the latter momentum conservation that further bypasses the subtle dynamic reciprocity and enables desirable isolation for backward-traversing noise. Alternatively, this observation plays an essential role in our experimental demonstrations. The bandwidth (∼3 MHz) of the parametric gain for the signal field is largely determined by the dispersion as well as the circulating optical power inside the microcavity, and it is relatively narrower than the cavity resonance linewidth in our experiment^27^,^28^. To keep the system stable, optical non-reciprocity is demonstrated with the injected pump power below the threshold of the optical parametric oscillation. We note that optical parametric oscillation and its enabled Kerr frequency comb in high-*Q* microcavities have been studied in previous studies^29^,^30^. It is worth emphasizing that another critical procedure of our experiment is to fabricate the sample with almost no backscattering^31^ for both pump and signal waves. This is because the backscattering will severely impair the desired directionality of the momentum conservation.

### Experimental designs

**Figure 1:**
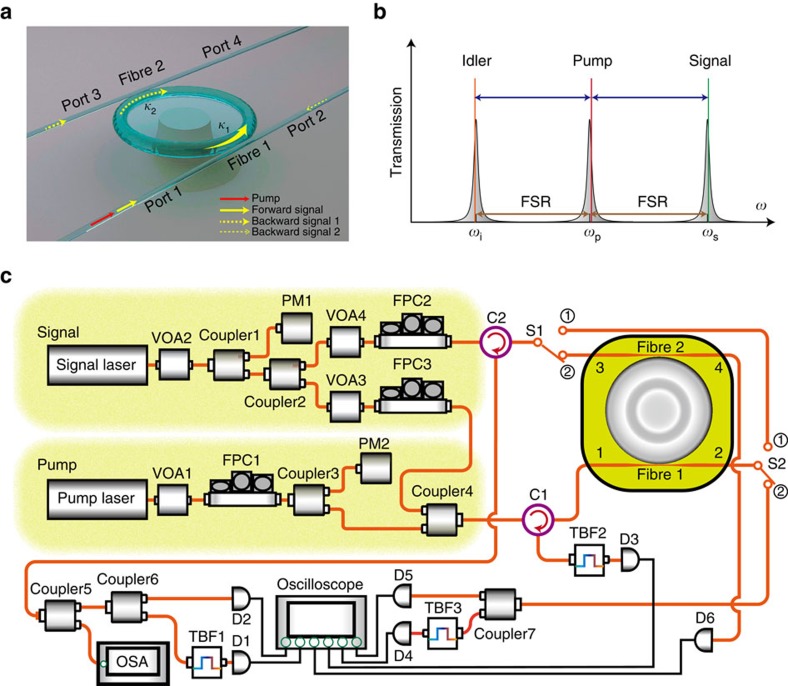
Optical isolator with parametric amplification in a high-*Q* microtoroid cavity. (**a**) 3D schematic of forward (solid yellow arrow) and backward (dashed yellow arrow) propagation configurations based on signal inputs at ports 1 and 3 in the two-fibre-coupling case (at ports 1 and 2 in the single-fibre-coupling case). (**b**) Frequency spectral representation of the pump, signal and idler waves involved in four-wave mixing, whose occurrence only appears in the forward direction due to phase matching. The red line represents the continuous-wave pump laser. The green and yellow lines denote, respectively, the parametric-amplified forward signal and generated idler waves. (**c**) Schematic of the experimental setup. C, optical fibre circulator; D, photodetector; FPC, fibre polarization controller; OSA, optical spectrum analyser; PM, power metre; S, optical switch; TBF, tunable bandpass filter; VOA, variable optical attenuator.

As schematically illustrated in Fig. 1a and Supplementary Fig. 1, our design consists of a high-*Q* silica whispering-gallery-mode microtoroid^26^ fabricated on a silicon chip and evanescently coupled to two tapered optical fibres (labelled as fibre 1 and fibre 2). The experimental setup (see Methods for details) is depicted in Fig. 1c, where the forward signal beam was seeded from port 1; while the backward signal was launched from either port 3 (in the two-fibre-coupling case) or port 2 (in the single-fibre-coupling case). The pump laser was always input through port 1. In the experiment, we first chose the two-fibre-coupled structure to interrogate optical isolation induced by phase-matched parametric amplification, as this structure permits versatile controllability and easily illustrates the non-reciprocal property. The pump and signal modes (shown in Fig. 1b) are properly selected for the microtoroid cavity with *Q* factors of 8.03 × 10^7^ and 7.40 × 10^7^ at the wavelengths of 1562.93 and 1557.39 nm, respectively. The wavelength difference between the pump and signal fields is about 5.54 nm, coinciding with the free spectral range of the microcavity. The idler is also generated one free spectral range away from the pump mode, which makes it easier to be separated and filtered out from the pump and signal after port 2.

### Optical isolation with bidirectional signal injections

**Figure 2:**
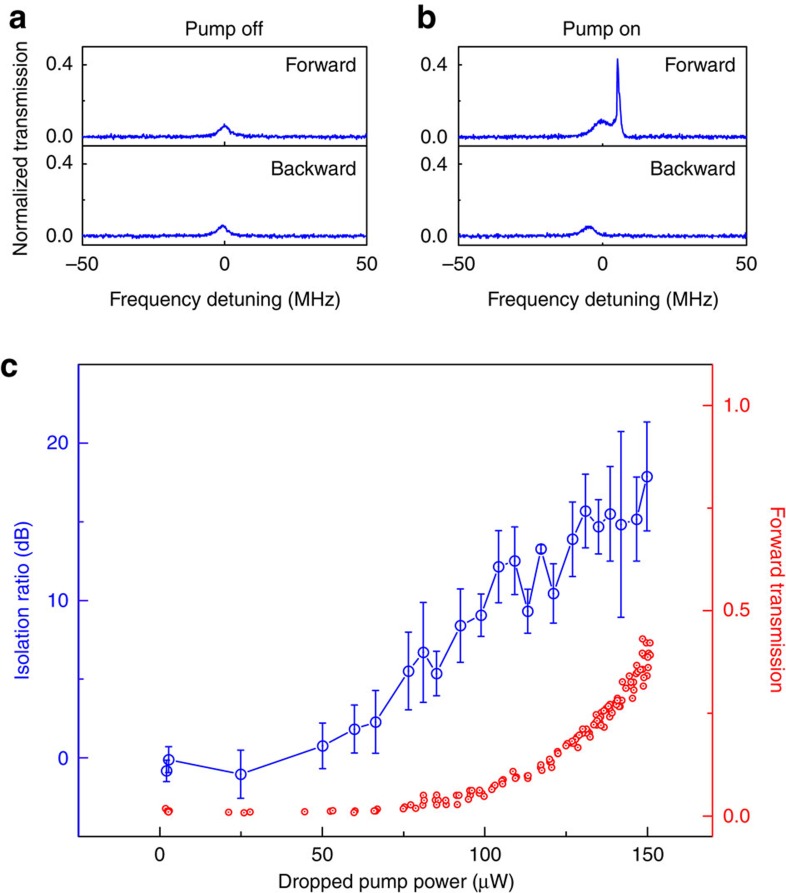
Optical isolation versus dropped pump power in the two-fibre-coupling case. (**a**) With pump off, as expected, reciprocal transmission is obtained. The single peak appearing in both forward and backward transmission spectra indicates negligible backscattering effect in the microcavity. (**b**) With pump on, typical asymmetric transmission spectra are observed when the parametric gain compensates the loss. The sharp peak in forward transmission marks the centre of the parametric gain. (**c**) Measured optical isolation ratio as a function of the dropped pump power (blue circles). In contrast, the normalized forward signal transmission (red circles) bears with a smooth behaviour. The error bars are the standard deviation of the measured isolation ratios under same pump conditions, which suggest the isolation uncertainty be mostly owing to the backward transmission. Parameters: the simultaneous presence of equal forward and backward signal power is 5.6 μW, *κ*_1_=2π × 1.95 MHz and *κ*_2_=2π × 0.18 MHz.

To test the feasibility of our methodology, we begin with the verification of reciprocal transmission of the forward and backward signal light by turning the pump light off. As expected, reciprocal transport (Fig. 2a) is recorded whenever the microtoroid is subjected to the forward and backward signal input simultaneously or separately. The single-peak transmission spectrum observed in both directions (Fig. 2a) also confirms the absence of the backscattering inside the cavity. We then switch on the pump laser and thermally lock it to the microcavity to produce parametric gain for the seeded signal light. Thanks to the phase-matched parametric amplification, the forward signal now returns more output from port 3 than the backward signal as long as the gain compensates the loss. Typical non-reciprocal transmission spectra are presented in Fig. 2b, where the sharp peak appearing near the spectral centre of the forward original input symbolizes the maximal location of the parametric gain (also see Supplementary Fig. 2a). Similar to other nonreciprocal light transmission enabled by resonant structures, the performance of our isolator can be well controlled by tuning a series of system parameters such as the optical coupling rates (*κ*_1_, *κ*_2_) and the dropped pump power (*P*_p_, the power injected into the microcavity). Figure 2c shows the measurement of non-reciprocal signal transmission as a function of *P*_p_. Its trend indicates that the isolation ratio (blue circles) grows from 0 to ∼18 dB by gradually increasing *P*_p_. The relatively large error fluctuations are mainly due to the large uncertainty from the backward transmitted-signal measurement. This is further confirmed by the stable output in the forward configuration (red circles). During the process, the incident forward and backward signal beams were maintained with equal power of 5.6 μW, and the coupling rate *κ*_1_ (*κ*_2_) between fibre 1 (2) and the toroid was set to 2π × 1.95 MHz (2π × 0.18 MHz). Note again that, to the best of our knowledge, there is no direct measurement on a chip-based optical isolator with the simultaneous presence of the signal light from both directions so far. More importantly, the acquired isolation here evidently implies that the current scheme is not limited by the dynamic reciprocity.

**Figure 3:**
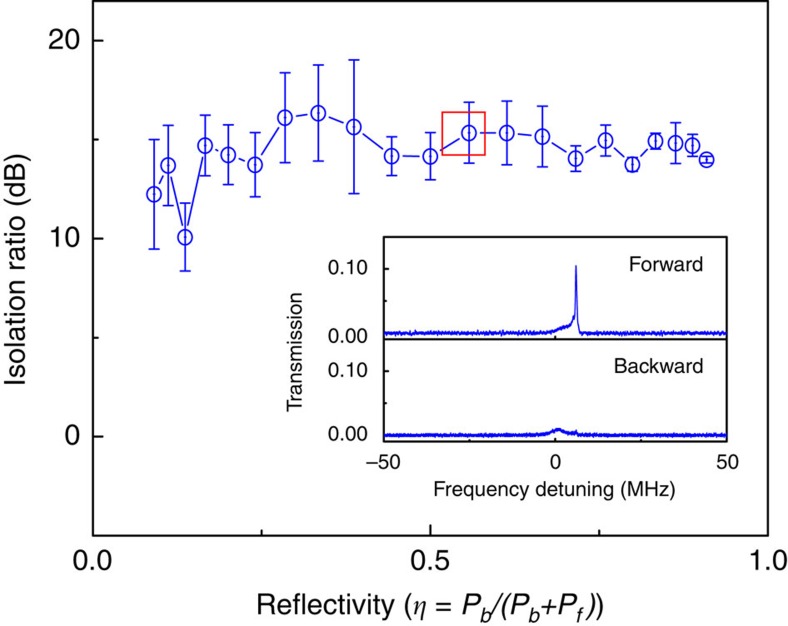
Optical isolation in terms of reflectivity in the two-fibre-coupling case. The backward input signal power *P*_b_ is increased from 95.57 nW to 9.57 μW. The dropped pump power is maintained at 78 μW. The inset shows the transmission spectra of the marked point with *P*_b_=1.20 μW. The error bars are the standard deviation of the measured optical isolations under the same parameter conditions, whose fluctuations are mainly owing to the backward signal transmission. Other parameters: the fixed forward input signal power is *P*_f_=0.97 μW, *κ*_1_=2π × 0.29 MHz and *κ*_2_=2π × 0.05 MHz.

Figure 3 is a plot of the isolation behaviour versus the reflectivity. Here, the reflectivity is referred to as the fraction of the backward input signal power (*P*_b_) over the total input signal power in both directions, 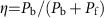. In the experiment, the pump wavelength was thermally locked to the cavity mode with a fixed detuning. The input pump power was about 451.20 μW, while the power dropped into the cavity was kept at 78 μW. The input signal power in the forward direction was set at *P*_f_=0.97 μW, while *P*_b_ increased gradually from 95.57 nW to 9.57 μW (see Supplementary Note 2). Owing to the contamination in the experiment, the optical *Q*-factors corresponding to the pump and signal modes reduce to 4.20 × 10^7^ and 5.79 × 10^7^. Remarkably, reliable isolation with a ratio well above 10 dB is readily available for *η* between 0 and 1. The inset is a snapshot of the typical transmission spectra obtained in the forward and backward directions. To further evaluate the device performance, we have investigated the non-reciprocity in terms of the input signal powers under the condition of *P*_b_=*P*_f_ (see Supplementary Fig. 3). In this case, we fix all other parameters but only alter input powers of both forward and backward signal light. Apparent isolation can be well held with a ratio above 15 dB for *P*_b_=*P*_f_ in the range of 0.1–10 μW (see Supplementary Note 2). In addition, the typical insertion loss as low as ∼4 dB is achieved throughout the measurement (for example, Fig. 2).

## Discussion

**Figure 4:**
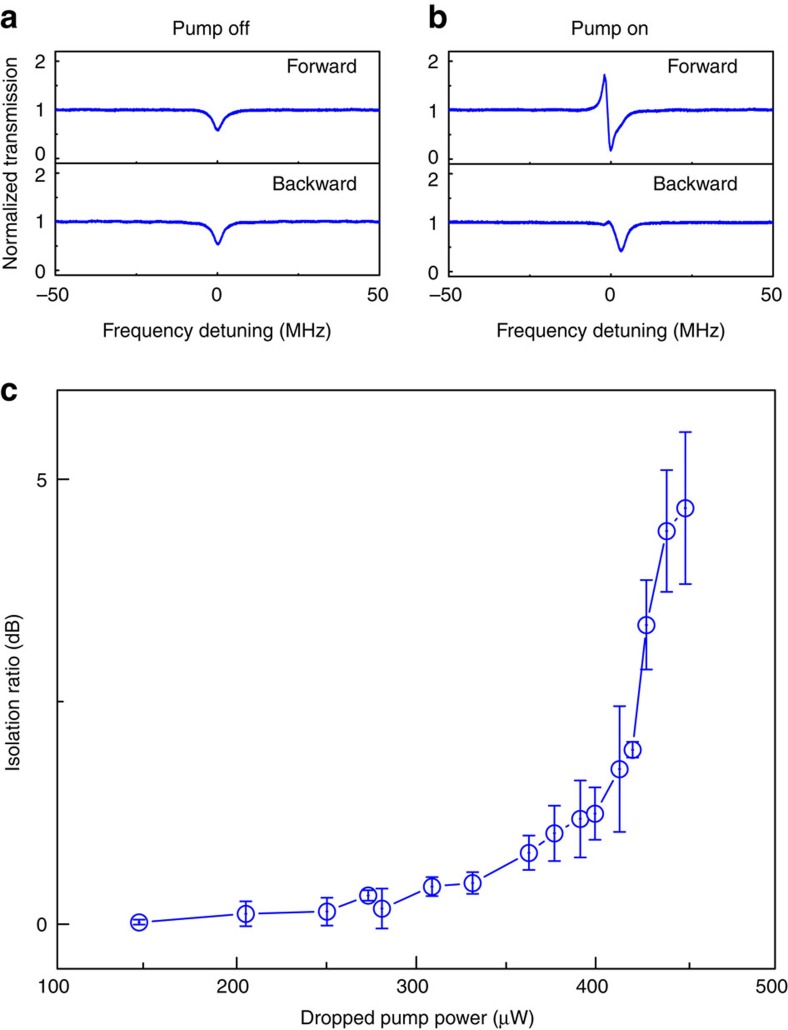
Optical non-reciprocity versus dropped pump power in the single-fibre-coupling case. (**a**) Reciprocal transmission is expected with pump off. (**b**) With pump on, typical non-reciprocal transmission spectra are recorded as the forward signal experiences phase-matched parametric amplification but the backward does not. (**c**) The optical isolation is characterized only as a function of the dropped pump power. The error bars represent the standard deviations of measured isolation uncertainties under the same conditions, and their fluctuations are mainly affected by the uncertainties of backward signal transmission. Parameters: the simultaneously injected forward and backward signal powers are equal to 1.46 μW and *κ*_1_=2π × 0.4 MHz.

Unlike previous demonstrations^17^,^21^,^23^ (relying highly upon a microresonator asymmetrically coupled to two waveguides), optical non-reciprocity induced by the parametric amplification here can even be implemented with only a single-fibre coupling. This can be understood from the directionality of phase matching specified by the pump-photon momentum, as it resembles an external magnetic field applied in the common magneto-optical effect. The experimental proof is performed via removing fibre 2 (Fig. 1a,c). Using another sample with optical *Q*-factors of 2.07 × 10^7^ and 5.69 × 10^7^ at the pump and signal modes, we first confirmed reciprocal transmission of signal fields by switching off the pump laser. The experimental data are shown in Fig. 4a, where forward and backward input signals have equal power of 1.46 μW. By turning the pump on, the non-reciprocity becomes more appreciable as the parametric gain is large enough in the cavity. The isolation trend versus the dropped pump power is displayed in Fig. 4c. As one can see, when the dropped pump power is greater than 350 μW (also see Supplementary Fig. 2b), asymmetric transmission starts to become distinct. The typical output spectra measured at ports 2 and 1 are presented in Fig. 4b. Again, the peak arising in the forward transmission spectrum comes from the parametric amplification. Besides, with the same setting we have confirmed asymmetric transmission as usual by launching the signal field only in either the forward or the backward direction but never in both (Supplementary Fig. 4).

In summary, in this work we have conceptually demonstrated the possibility of designing and implementing an optical isolator by employing phase-matched parametric amplification in a chip-based high-*Q* microtoroid resonator. The simple scheme, operated under practical conditions, explicitly proves itself to be useful for protecting a laser from harmful reflections, which is one of the most important applications of non-reciprocal devices. We note that our methodology shares certain similarities with the schemes of stimulated Brillouin scattering^21^, Bragg scattering four-wave-mixing^22^ and acousto-optics^24^ in the sense that they are linear in their response with respect to the signal field^25^. From this point of view, these nonlinearity-based optical isolators could be considered as linear ones for the signal beam, and are anticipated not to be limited by the dynamic reciprocity, as noted in ref. 25. Another significant aspect of this design is its ability to have low insertion loss available with the gain amplification. Because the device exploits cavity resonance enhancement and phase-matched parametric amplification, the main drawback of the current work is its working range limited to narrow bandwidth in nature. By employing the optical microcavity with a larger nonlinear-refractive-index material^32^ (such as silicon nitride or high-index doped silica) and a lower optical quality factor, this isolation bandwidth could, in principle, be broadened. By using other suitable platforms such as waveguides, the operating isolation bandwidth could be substantially broadened, but inevitable propagation loss may lead to large insertion loss. Also, it is possible to integrate a waveguide to a silica microcavity without sacrificing isolation performance by replacing the tapered fibres with integrated waveguides^33^,^34^. Nevertheless, it is expected that the current device could be potentially useful for integrated quantum photonics using narrowband single photons or continuous variables as information carriers. Importantly, our results convey that it is feasible to build a non-magnetic optical isolator using nonlinear optics for laser protection and optical information processing in integrated photonics. In addition, we anticipate that our work could stimulate more efforts on identifying and developing practical and magnetic-free non-reciprocal devices utilizing nonlinear means.

## Methods

### Optical non-reciprocity measurements

Figure 1c presents a schematic diagram of the experimental setup for isolation measurements on the proposed nonlinear optical isolator. The setup looks similar to our previous works^17^,^19^, except that it is further capable of simultaneous injections of signal fields in both forward and backward directions. By adjusting two optical switches S1 and S2, the system is interchangeable between two coupling cases: single-fibre-coupling and two-fibre-coupling. The polarizations of the forward and backward fields are, respectively, adjusted by FPC3 and FPC2 to match the polarizations supported by the cavity modes. Experimentally, two narrow linewidth continuous-wave tunable lasers operating in the 1,550 nm band are used as a pump and signal fields, respectively. During the optical isolation measurement, the pump cavity mode is thermally locked to the pump laser and the dropped pump power is adjusted by controlling the pump frequency detuning with respect to the cavity resonance frequency.

### Two-fibre-coupling case

For the experiment with the two-fibre-coupled structure, S1 and S2 were switched into . The forward signal was launched into the microcavity through port 1 and was extracted at port 3. After passing an optical fibre circulator (C2), two optical couplers (Coupler5 and Coupler6), and a tunable bandpass filter (TBF1), the forward signal emitted from port 3 is measured by a photodetector (D1). The forward signal exiting from port 2 was routed to S2, Coupler7, TBF3 and D4 for measurement. Correspondingly, the backward signal was injected from port 3 and extracted from the cavity from port 1 followed by C1, Filter2 and D3 for analysis. D6 was used to measure the coupling rate between fibre 2 and the microcavity. During the measurement, the pump field was always launched from port 1 and its transmission at port 2 was detected by D5 after traversing S2 and Coupler7.

### Single-fibre-coupling case

By switching S1 and S2 into , as schematically shown in Fig. 1c, the system is in the single-fibre-coupling case. That is, the microtoroid resonator is only coupled with fibre 1. Fibre 2 was moved away via a nano-positioner. The forward signal beam was launched into the toroid through port 1 and its output from port 2 was directed to S2, S1, C2, Coupler5, Coupler6, TBF1 and D1 for detection. The backward signal light was incident through port 2 and its output from port 1 was directed to C1, TBF2 and D3 for measurement. The transmitted pump light from port 2 was measured by D2 after passing S2, S1, Coupler5 and Coupler6.

For the experiment on investigating non-reciprocal transmission by launching the signal either in the forward configuration or the backward as done in previous research^14^,^15^,^16^,^17^,^18^,^19^,^20^,^21^,^22^,^23^,^24^,^25^, on the other hand, one only needs to switch off one path of the signal propagation through a variable optical attenuator (VOA 4 or VOA3).

### Data availability

The data that support the findings of this study are available from the corresponding authors on request.

## Additional information

**How to cite this article:** Hua, S. *et al*. Demonstration of a chip-based optical isolator with parametric amplification. *Nat. Commun.* **7,** 13657 doi: 10.1038/ncomms13657 (2016).

**Publisher's note**: Springer Nature remains neutral with regard to jurisdictional claims in published maps and institutional affiliations.

## Supplementary Material

Supplementary Information Supplementary Figures 1-4, Supplementary Notes 1-2 and Supplementary References
