# Supplementary material for: Demonstration of a chip-based optical isolator with parametric amplification
Source: Nat Commun. 2016 Nov 25;7:13657. doi: 10.1038/ncomms13657 (PMC5133705; doi:10.1038/ncomms13657)
Supplement: Supplementary Information — Supplementary Figures 1-4, Supplementary Notes 1-2 and Supplementary References [file ncomms13657-s1.pdf]

# Supplementary Figures

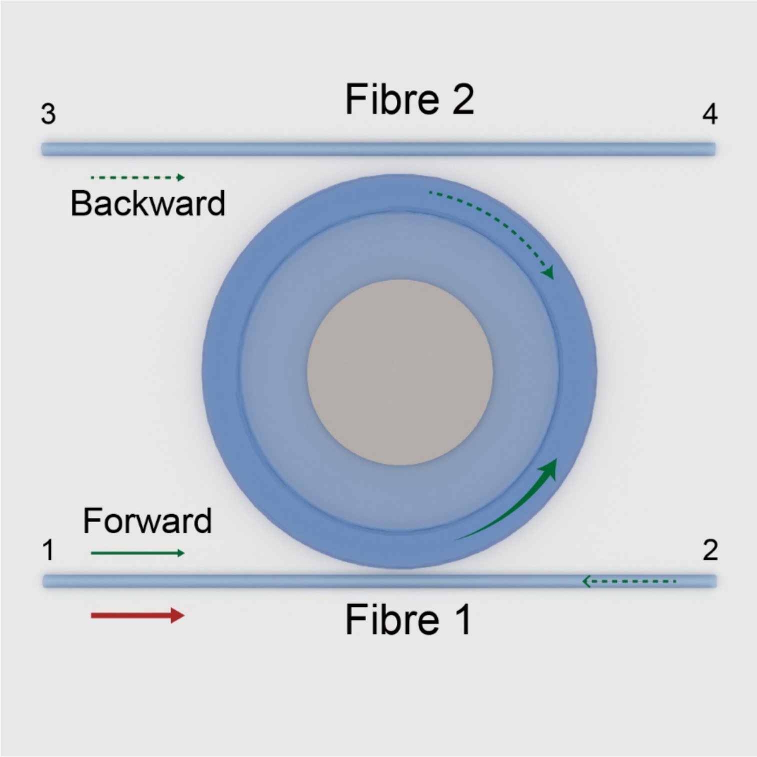


**Supplementary Figure 1: Sketch of demonstration of chip-based optical nonreciprocity.** The optical isolation is studied in two cases: In the two-fibre-coupling case where the high-$Q$ WGM microtoroid is coupled with both fibres 1 and 2, the forward (backward) direction is defined as the input signal field from port 1 (port 3); while in the single-fibre-coupling case where the microtoroid cavity is only coupled with fibre 1, the forward (backward) direction is defined as the input signal field from port 1 (port 2). In both cases, the input pump laser is always injected from port 1.

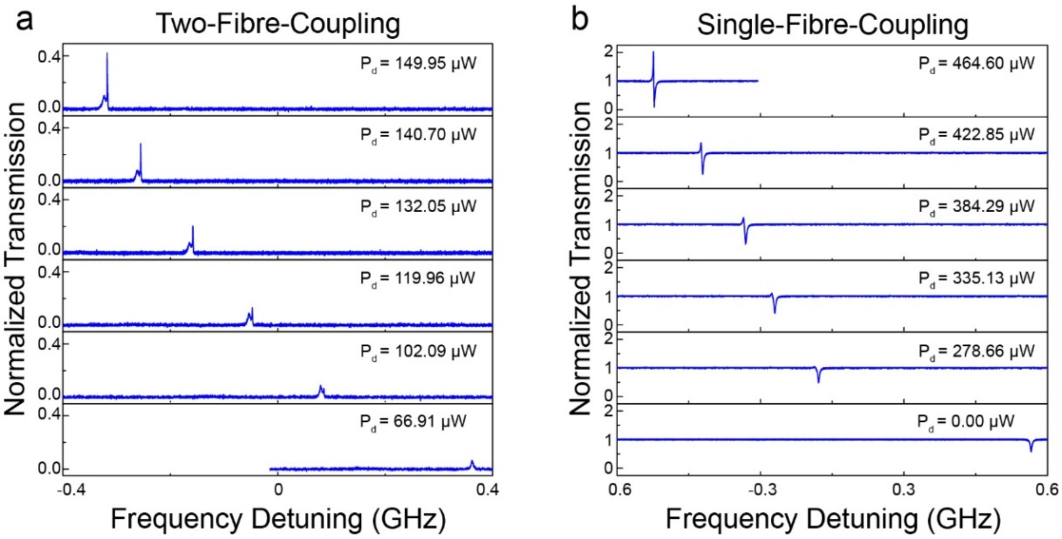


**Supplementary Figure 2: Typical normalized transmission spectra of the forward signal field measured with two-fibre (a) and single-fibre (b) coupling.** Due to the thermal effect induced by the circulating pump power inside the microcavity, in both cases the wavelength redshift of the signal mode is observed as increasing the dropped pump power. Using the theory developped in Supplementary Note 1, we have numerically confirmed these spectral profiles by plugging in the experimental parameters.

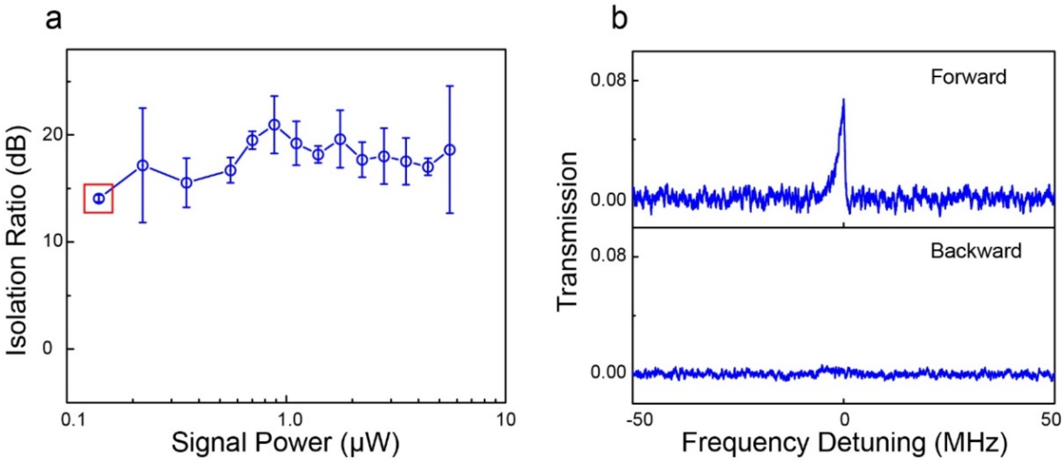


**Supplementary Figure 3:** (**a**) **Isolation ratio versus input signal power in the two-fibre-coupling case.** Here the signal inputs simultaneously enter the cavity from both directions and are set with equal power for each measurement. The isolation performance is characterized within a range of signal powers by fixing the coupling rates ($\kappa_1 = 2\pi \times 0.29$ MHz and $\kappa_2 = 2\pi \times 0.04$ MHz) between the microcavity and two microfibers and the dropped pump power of 78 μW. The experimental data reveal high isolation ratio (of above 15 dB) available for the working signal input power from 139.89 nW to 6.5 μW. (**b**) The normalized transmission spectra of the marked point in (**a**) with the forward and backward signal power of ~139.89 nW.

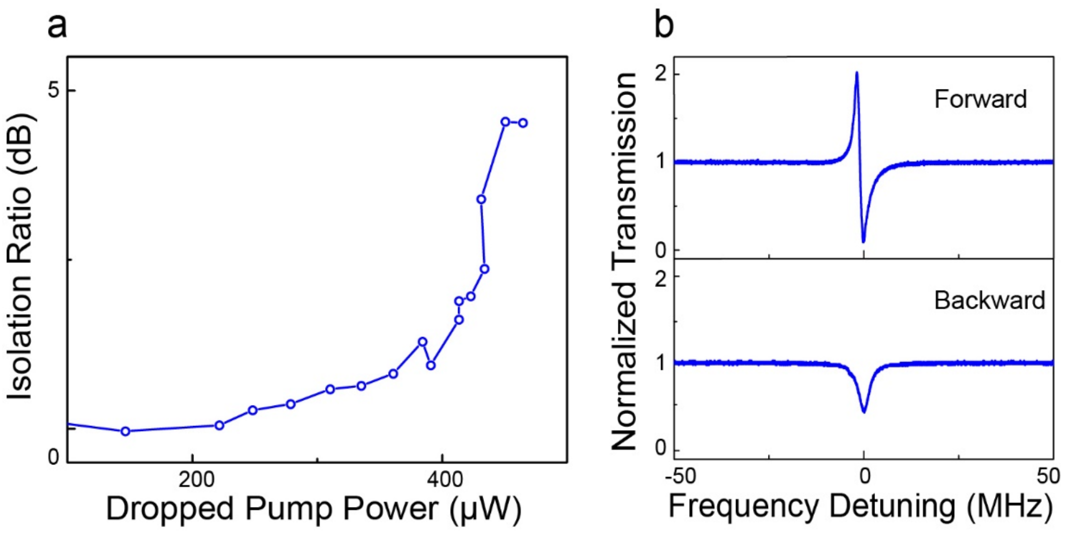


**Supplementary Figure 4: Nonreciprocal transmission versus the dropped pump power in the single-fibre-coupling case.** Here the signal field is launched in either the forward or the backward direction but never in both. Such a procedure has been adopted in all reported isolation experiments up till today. (**a**) The isolation ratio is measured only as a function of the dropped pump power by fixing all other parameters. (**b**) Nonreciprocal signal transmission spectrum obtained typically in the forward or backward direction. In comparison with Fig. 4 in the main text, one can see that both operating situations yield very similar nonreciprocal transmission behaviour. The implementations with both simultaneous and separate injections of signal fields in two directions unambiguously prove our design to be capable of a real isolator for laser protection, one of the most important applications of nonreciprocal devices. All the parameters here are the signal power of 1.46 μW and $\kappa_1 = 2\pi \times 0.4$ MHz, same as those in Fig. 4 in the main text.

# Supplementary Notes

## Supplementary Note 1: Optical parametric amplification in a WGM microcavity

Our scheme explores phase-matched parametric amplification in a whispering-gallery-mode (WGM) microresonator with high-$Q$ factors on a silicon chip. Due to the inversion symmetry of silica, the lowest-order nonlinearity is the third-order nonlinearity which leads to the elemental parametric interaction, four-wave mixing (FWM), to convert two pump photons $(\omega_{\mathrm{p}}, \mathbf{k_p})$ into signal $(\omega_{\mathrm{s}}, \mathbf{k_s})$ and idler $(\omega_{\mathrm{i}}, \mathbf{k_i})$ photons (Fig. 1b in the main text). In order for parametric amplification to occur, the conservations of both energy and momentum (i.e. phase matching) must be preserved in the FWM process. We note that by manipulating the Kerr nonlinearity, optical parametric oscillation[1] and optical frequency combs[2] have been experimentally realized in high-$Q$ silica WGM microtoroid cavities. In these two works, momentum is intrinsically conserved as signal and idler angular mode numbers ($l$) are symmetric with respect to the pump mode by following $l_{\mathrm{s,i}} = l_{\mathrm{p}} \pm M$, while energy conservation, $2\omega_{\mathrm{p}} = \omega_{\mathrm{s}} + \omega_{\mathrm{i}}$, is satisfied by controlling the cavity dispersion including both material dispersion and geometry dispersion. In our experiment, the geometry dispersion is engineered by properly controlling the major and minor diameters of the microtoroids[3]. In contrast to the optical parametric oscillation process[1,2], the process of optical parametric amplification involved in our experiment demands injecting both the co-propagating pump and signal modes into the microcavity, which result in the momentum to be favorably preserved along with the co-propagation direction, i.e., the forward configuration. It is this directionality of momentum conservation that plays an essential role in bypassing the dynamic reciprocity[4] suffered by Kerr or Kerr-type nonlinearities. To fulfill such directionality, the sample should be devoid of backscattering[5], although it is commonly present in high-$Q$ WGM microcavities. These two elements are interrelated with and indispensable of each other. It is worth mentioning that to stabilize the system, optical isolation here is implemented by keeping the pump power below the threshold of the optical parametric oscillation.

Since the optical parametric amplification is more favourable in the forward direction through FWM and the signal light is weaker than the pump, the process involving pump, signal and idler field amplitudes $(A_{\mathrm{p}}, A_{\mathrm{s}}^{\mathrm{f}}, A_{\mathrm{i}})$ can be described by the simplified Langevin equations according to Refs.[6,7],

$$\begin{cases} \frac{dA_{\mathrm{p}}}{dt} = -\Gamma_{\mathrm{p}} A_{\mathrm{p}} + ig\left|A_{\mathrm{p}}\right|^2 A_{\mathrm{p}} + 2igA_{\mathrm{p}}^* A_{\mathrm{s}}^{\mathrm{f}} A_{\mathrm{i}} + \sqrt{\kappa_{\mathrm{ep}}^{(1)}} S_{\mathrm{p}}, \\ \frac{dA_{\mathrm{s}}^{\mathrm{f}}}{dt} = -\Gamma_{\mathrm{s}} A_{\mathrm{s}}^{\mathrm{f}} + 2ig\left|A_{\mathrm{p}}\right|^2 A_{\mathrm{s}}^{\mathrm{f}} + igA_{\mathrm{p}}^2 A_{\mathrm{i}}^* + \sqrt{\kappa_{\mathrm{es}}^{(1)}} S_{\mathrm{s}}^{\mathrm{f}}, \\ \frac{dA_{\mathrm{i}}}{dt} = -\Gamma_{\mathrm{i}} A_{\mathrm{i}} + 2ig\left|A_{\mathrm{p}}\right|^2 A_{\mathrm{i}} + igA_{\mathrm{p}}^2 \left(A_{\mathrm{s}}^{\mathrm{f}}\right)^*, \end{cases} \tag{1}$$

where $\Gamma_{\mathrm{p}} = i\left(\omega_{\mathrm{p}} - \bar{\omega}_{\mathrm{p}}\right) + \frac{\kappa_{\mathrm{p}}}{2} + \frac{\kappa_{\mathrm{ep}}}{2}$, $\Gamma_{\mathrm{s}} = i(\omega_{\mathrm{s}} - \bar{\omega}_{\mathrm{s}}) + \frac{\kappa_{\mathrm{s}}}{2} + \frac{\kappa_{\mathrm{es}}}{2}$, $\Gamma_{\mathrm{i}} = i(\omega_{\mathrm{i}} - \bar{\omega}_{\mathrm{i}}) + \frac{\kappa_{\mathrm{i}}}{2} + \frac{\kappa_{\mathrm{ei}}}{2}$

with $\omega_m$, $\bar{\omega}_m$, $\kappa_m$, and $\kappa_m = \kappa_{em}^{(1)} + \kappa_{em}^{(2)}$ $(m = p, s, i)$ the frequency of the cavity mode, the

carrier frequency of the pump, signal or the generated light, the intrinsic decay rate of the optical $m$ mode, and the external coupling rate to the two fibres, respectively, and * denotes the complex conjugate. $S_\mathrm{p}$ and $S_\mathrm{s}^\mathrm{f}$ stand for, respectively, the input power amplitudes of the forward pump and signal fields. $g = \hbar\omega_\mathrm{p}^2 c n_2 n_0^{-2} V_\mathrm{eff}^{-1}$ is the nonlinear coupling coefficient, where $n_2 = 2.2 \times 10^{-20}$ m$^2$W$^{-1}$ is the Kerr nonlinearity of silica[8], $n_0$ is the silica refractive index, $V_\mathrm{eff}$ is the effective mode volume of optical mode in cavity, and $c$ is the speed of light in vacuum.

We seek solutions of the set of equations (1) in the steady-state approximation. After some algebra, the amplitude of the forward signal field inside the microcavity is

$$A_\mathrm{s}^\mathrm{f} = \frac{-\sqrt{\kappa_\mathrm{es}^{(1)}}S_\mathrm{s}^\mathrm{f}\left(\Gamma_\mathrm{i}^* + 2ig|A_\mathrm{p}|^2\right)}{\left(-\Gamma_\mathrm{s} + 2ig|A_\mathrm{p}|^2\right)\left(\Gamma_\mathrm{i}^* + 2ig|A_\mathrm{p}|^2\right) + g^2|A_\mathrm{p}|^4}. \tag{2}$$

According to the coupled-mode theory[9], the amplitude of the transmitted signal field at port 3 (see Supplementary Fig. 1) takes the form of,

$$S_\mathrm{s3}^\mathrm{f} = \sqrt{\kappa_\mathrm{es}^{(2)}}A_\mathrm{s}^\mathrm{f} = \frac{-\sqrt{\kappa_\mathrm{es}^{(1)}\kappa_\mathrm{es}^{(2)}}S_\mathrm{s}^\mathrm{f}\left(\Gamma_\mathrm{i}^* + 2ig|A_\mathrm{p}|^2\right)}{\left(-\Gamma_\mathrm{s} + 2ig|A_\mathrm{p}|^2\right)\left(\Gamma_\mathrm{i}^* + 2ig|A_\mathrm{p}|^2\right) + g^2|A_\mathrm{p}|^4}. \tag{3}$$

For the backward propagating signal, on the contrary, as the available phase-matched parametric amplification is not present, the backward signal-field amplitude inside the microresonator evolves as

$$\frac{dA_\mathrm{s}^\mathrm{b}}{dt} = -\Gamma_\mathrm{s}A_\mathrm{s}^\mathrm{b} + 2ig|A_\mathrm{p}|^2 A_\mathrm{s}^\mathrm{b} + \sqrt{\kappa_\mathrm{es}^{(2)}}S_\mathrm{s}^\mathrm{b}. \tag{4}$$

As a result, Eqn. (4) yields the backward signal transmission spectrum at port 1 as

$$S_\mathrm{s1}^\mathrm{b} = \sqrt{\kappa_\mathrm{es}^{(1)}}A_\mathrm{s}^\mathrm{b} = \frac{\sqrt{\kappa_\mathrm{es}^{(1)}\kappa_\mathrm{es}^{(2)}}S_\mathrm{s}^\mathrm{b}}{\Gamma_\mathrm{s} - 2ig|A_\mathrm{p}|^2}. \tag{5}$$

In terms of the scattering matrix[10], Eqns. (3) and (5) can be recast into the following form

$$\begin{pmatrix} S_\mathrm{s1}^\mathrm{b} \\ S_\mathrm{s3}^\mathrm{f} \end{pmatrix} = \begin{bmatrix} 0 & \dfrac{\sqrt{\kappa_\mathrm{es}^{(1)}\kappa_\mathrm{es}^{(2)}}}{\Gamma_\mathrm{s} - 2ig|A_\mathrm{p}|^2} \\ \dfrac{-\sqrt{\kappa_\mathrm{es}^{(1)}\kappa_\mathrm{es}^{(2)}}\left(\Gamma_\mathrm{i}^* + 2ig|A_\mathrm{p}|^2\right)}{\left(-\Gamma_\mathrm{s} + 2ig|A_\mathrm{p}|^2\right)\left(\Gamma_\mathrm{i}^* + 2ig|A_\mathrm{p}|^2\right) + g^2|A_\mathrm{p}|^4} & 0 \end{bmatrix} \begin{pmatrix} S_\mathrm{s}^\mathrm{f} \\ S_\mathrm{s}^\mathrm{b} \end{pmatrix}. \tag{6}$$

Two conclusions can be directly drawn from Eqn. (6): On the one hand, because of the directional phase matching, optical nonreciprocity is readily established regardless of simultaneous injections of both forward and backward signal inputs or separate signal injections. To bypass the dynamic reciprocity, on the other, here it requires negligible backscattering within the microcavity for both pump and signal waves, such that no appreciable phase-matched parametric amplification occurs for the backward input signal wave. For this purpose, the microtoroid cavities are prepared with 6 μm-thick thermal oxide film to form relatively large mode volumes. In the experiment, the major diameter of the fabricated microtoroid is ~96 μm while the minor diameter is ~13 μm. Similar to the previous work[11] on generating Kerr frequency combs, here the microtoroid also operates in the normal dispersion region.

Similarly, for the single-fibre-coupling case (i.e., the microcavity is only coupled with

fibre 1), one can obtain the outputs at port 2 and port 1 (see Supplementary Fig. 1) as

$$S_{s1}^{b} = \sqrt{\kappa_{es}^{(1)}} A_s^{b} - S_s^{b}, \text{ and } S_{s2}^{f} = \sqrt{\kappa_{es}^{(1)}} A_s^{f} - S_s^{f}. \quad (7)$$

In terms of the scattering matrix[10], Eqn. (7) becomes

$$\begin{pmatrix} S_{s1}^{b} \\ S_{s2}^{f} \end{pmatrix} = \begin{bmatrix} 0 & \frac{\kappa_{es}^{(1)}}{\Gamma_s - 2ig|A_p|^2} - 1 \\ \frac{-\kappa_{es}^{(1)}\left(\Gamma_i^* + 2ig|A_p|^2\right)}{\left(-\Gamma_s + 2ig|A_p|^2\right)\left(\Gamma_i^* + 2ig|A_p|^2\right) + g^2|A_p|^4} - 1 & 0 \end{bmatrix} \begin{pmatrix} S_s^{f} \\ S_s^{b} \end{pmatrix}. \quad (8)$$

It is apparent from the off-diagonal elements in Eqn. (8) that both Lorentz reciprocity and dynamic reciprocity break down. Alternatively, Eqn. (8) is applicable to describe light transport for both cases with simultaneously and separately launching forward and backward signal lasers.

The isolation ratio is computed by following its definition. That is,

$$\text{Isolation Ratio (dB)} \equiv 10 \times \log_{10} \frac{\text{Maximum of } T^{f}}{\text{Transmission at corresponding frequency}},$$

where the normalized transmission spectra, $T^{f}$, in two different coupling cases are defined as,

$$T^{f} = \left| \frac{-\sqrt{\kappa_{es}^{(1)}\kappa_{es}^{(2)}}\left(\Gamma_i^* + 2ig|A_p|^2\right)}{\left(-\Gamma_s + 2ig|A_p|^2\right)\left(\Gamma_i^* + 2ig|A_p|^2\right) + g^2|A_p|^4} \right|^2,$$ for the two-fiber-coupling case; while

$$T^{f} = \left| \frac{-\kappa_{es}^{(1)}\left(\Gamma_i^* + 2ig|A_p|^2\right)}{\left(-\Gamma_s + 2ig|A_p|^2\right)\left(\Gamma_i^* + 2ig|A_p|^2\right) + g^2|A_p|^4} - 1 \right|^2,$$ for the single-fiber-coupling case.

## Supplementary Note 2: Self-heating effect

One interesting and important issue related to the current work is the presence of the self-heating effect. It is known that the optical absorption inside the ring cavity results in a temperature change, and this temperature change leads to the wavelength shift of the cavity resonance due to the backaction. Although the portion absorbed by the dielectric is very small, self-heating is non-negligible especially as the circulating power inside the cavity is extremely high because of its high $Q$ factor. In particular, the self-heating effect becomes obvious when the optical power within the cavity reaches above a certain level. In the current scheme, the self-heating originates mainly from two aspects: the input pump laser and the signal field of interest. One legitimate question is then how we manage the self-heating in our work.

a) Although the intrinsic optical $Q$-factor of the microcavity is close to $10^8$, due to the relatively large mode volume (we fabricated the microcavity using a 6-μm thick thermal oxide film instead of the 2-μm thick thermal oxide film typically used to fabricate a normal microtoroid) of the microtoroid used in the experiment, in the series of experiments reported here, we found that for the signal power below 10 μW, no appreciable self-heating effect is measurable throughout the measurements. This is ensured by checking if there is a thermal drifting of the resonance or not for the signal wave[12].
b) In contrast, the pump power is relatively high in the experiments. As a result, its

induced self-heating is inevitable in the experimental readouts. However, owing to the thermal effect in the microcavity, the cavity mode can be easily locked to the pump laser with a self-stabilization mechanism[12], and the microcavity temperature can be readily maintained to be stable. As such, the pump field only changes the temperature of the microcavity to a constant value, and it does not affect the experimental results essentially.

Although the performed isolation experiments were done for a signal power below 10 μW, a higher power operation range could be extended using a large-mode-volume or athermal microcavity. In such a case, caution should be taken to distinguish the thermo-optic effect from the FWM parametric amplification. Otherwise, as the signal power becomes much larger, more other nonlinear processes will be involved and so more investigation will be needed to address this issue accordingly. These might be of interest for future work.

# Supplementary References


1. Kippenberg, T. J., Spillane, S. M. & Vahala, K. J. Kerr-nonlinearity optical parametric oscillation in an ultrahigh-*Q* toroid microcavity. *Phys. Rev. Lett.* **93**, 083904 (2004).
2. Kippenberg, T. J., Holzwarth, R. & Diddams, S. A. Microresonator-based optical frequency combs. *Science* **332**, 555-559 (2011).
3. Del'Haye, P., Herr, T., Gavartin, E., Gorodetsky, M. L., Holzwarth, R. & Kippenberg, T. J. Octave spanning tunable frequency comb from a microresonator. *Phys. Rev. Lett.* **107**, 063901 (2011).
4. Shi, Y., Yu, Z. & Fan, S. Limitations of nonlinear optical isolators due to dynamic reciprocity. *Nature Photon.* **9**, 388-392 (2015).
5. Kippenberg, T. J., Spillane, S. M. & Vahala, K. J. Modal coupling in traveling-wave resonators. *Opt. Lett.* **27**, 1669-1671 (2002).
6. Matsko, A. B., Savchenkov, A. A., Strekalov, D., Ilchenko, V. S. & Maleki, L. Optical hyperparametric oscillations in a whispering-gallery-mode resonator: Threshold and phase diffusion. *Phys. Rev. A* **71**, 033804 (2005).
7. Chembo, Y. K. & Yu, N. Modal expansion approach to optical-frequency-comb generation with monolithic whispering-gallery-mode resonators. *Phys. Rev. A* **82**, 033801 (2010).
8. Boyd, R. W., *Nonlinear Optics*, 3rd Ed. (Academic Press, 2008).
9. Haus, H. A., in *Waves and fields in optoelectronics*, Ch. 7 (Prentice-Hall, 1984).
10. Jalas, D. *et al.* What is-and what is not-an optical isolator. *Nature Photon.* **7**, 579-582 (2013).
11. Del'Haye P., Schliesser, A., Arcizet, O., Wilken, T., Holzwarth, R. & Kippenberg, T. J. Optical frequency comb generation from a monolithic microresonator. *Nature* **450**, 1214 (2007).
12. Carmon, T., Yang, L. & Vahala, K. J. Dynamical thermal behavior and thermal self-stability of microcavities. *Opt. Express* **12**, 4742-4750 (2004).
